# Supplementary material for: FtsZ treadmilling is essential for Z-ring condensation and septal constriction initiation in Bacillus subtilis cell division
Source: Nat Commun. 2021 Apr 27;12:2448. doi: 10.1038/s41467-021-22526-0 (PMC8079713; doi:10.1038/s41467-021-22526-0)
Supplement: Supplementary file 3 — Description of Additional Supplementary Files [file 41467_2021_22526_MOESM3_ESM.docx]

# Supplementary Video LEGENDS

**Supplementary Video 1: FtsZ-GFP (SH130) organisation over cell cycle**. Representative video of FtsZ-GFP (SH130) dynamics, slow growth conditions (CDM, 30°C), imaged by ring-HILO microscopy with 1s exposure at 1 frame/ min. Video displayed at 20 frames per second (1200x actual speed). Scale bar, 1 μm.

**Supplementary Video 2: VerCINI imaging allows sensitive high-resolution imaging of division protein dynamics around the entire division septum for large numbers of cells.** Representative video of FtsZ-GFP (SH130) dynamics, imaged by VerCINI with ring-HILO illumination, 1s exposure and 2 second intervals. Unzoomed video section shows dynamics for all Z-rings recorded in a single VerCINI measurement. Zoomed video section highlights dynamics of 9 Z-rings in same measurement at higher magnification. Acquisitions displayed at 30 frames per second (60x actual speed).

**Supplementary Video 3: Exemplar results of VerCINI image processing on a nascent Z-ring for FtsZ-GFP (SH130) strain.** Representative video of FtsZ-GFP (SH130) dynamics, imaged continuously by VerCINI with ring-HILO illumination, 1s exposure. Left: Raw movie. Middle: Denoised and registered movie. Right: Movie after background subtraction using explicit model (Methods). Scale bar, 1 μm.

**Supplementary Video 4: FtsZ filaments in nascent Z-rings are sparse, highly dynamic and often static.** Representative videos of nascent FtsZ-GFP rings (SH130) imaged using VerCINI. Cells were imaged by ring-HiLO continuously with 1 s exposure. Acquisitions displayed at 15 frames per second(15x actual speed).Scale bar:1 µm.

**Supplementary Video 5: FtsZ filaments are predominantly treadmilling in mature/ early constricting rings**. Representative videos of mature/ early constricting FtsZ-GFP rings (SH130) imaged using VerCINI. Cells were imaged by ring-HiLO continuously with 1 s exposure. Acquisitions displayed at 15 frames per second (15x actual speed).Scale bar:1 µm.

**Supplementary Video 6: FtsZ filaments dynamics in late constricting rings are indistinguishable from those in mature/ early constricting rings.** Representative videos of mature/ early constricting FtsZ-GFP rings (SH130) imaged using VerCINI. Cells were imaged by ring-HiLO continuously with 1 s exposure. Acquisitions displayed at 15 frames per second (15x actual speed). Scale bar:1 µm.

**Supplementary Video 7: PC19 arrests FtsZ treadmilling within seconds throughout division.**  Representative videos of FtsZ-GFP (SH130) cells imaged using microfluidic VerCINI during instantaneous perturbation with excess PC19. Cells were imaged by ring-HiLO continuously with 1 s exposure. Acquisitions displayed at 15 frames per second (15x actual speed). Scale bar: 500 nm.

## Supplementary Video 8: PenG does not affect FtsZ treadmilling dynamics. Representative videos of FtsZ-GFP (SH130) cells imaged using microfluidic VerCINI during instantaneous perturbation with excess PenG. Cells were imaged by ring-HiLO continuously with 1 s exposure. Acquisitions displayed at 15 frames per second (15x actual speed). Scale bar: 500 nm.

## Supplementary Video 9: DMSO does not affect FtsZ treadmilling dynamics. Representative videos of FtsZ-GFP (SH130) cells imaged using microfluidic VerCINI during instantaneous perturbation with 1% DMSO. Cells were imaged by ring-HiLO continuously with 1 s exposure. Acquisitions displayed at 15 frames per second (15x actual speed). Scale bar: 500 nm.

## Supplementary Video 10: PC19 treatment prevents nascent Z-rings from condensing or constricting. FtsZ-GFP (SH130) cells imaged in a cellASIC device before and during treatment with excess PC19 in fast growth conditions (rich media 37^o^C). Representative nascent ring (red circle) tracked manually using TrackMate. Cells were imaged by ring-HiLO with 1 s exposure at 1 frame/min. Acquisitions displayed at 10 frames per second (600x actual speed).

## Supplementary Video 11: PC19 treatment prevents some mature Z-rings from constricting. FtsZ-GFP (SH130) cells imaged in a cellASIC device before and during treatment with excess PC19 in fast growth conditions (rich media 37^o^C). Representative mature ring (red circle) tracked manually using TrackMate. Cells were imaged by ring-HiLO with 1 s exposure at 1 frame/min. Acquisitions displayed at 10 frames per second (600x actual speed).

## Supplementary Video 12: Some mature Z-rings constrict post-PC19 treatment. FtsZ-GFP (SH130) cells imaged in a cellASIC device before and during treatment with excess PC19 in fast growth conditions (rich media 37^o^C). Representative mature ring (blue circle) tracked manually using TrackMate. Cells were imaged by ring-HiLO with 1 s exposure at 1 frame/min. Acquisitions displayed at 10 frames per second (600x actual speed).

## Supplementary Video 13: Constricting Z-rings typically continue constricting post-PC19 treatment. FtsZ-GFP (SH130) cells imaged in a cellASIC device before and during treatment with excess PC19 in fast growth conditions (rich media 37^o^C). Representative constricting ring (blue circle) tracked manually using TrackMate. Cells were imaged by ring-HiLO with 1 s exposure at 1 frame/min. Acquisitions displayed at 10 frames per second (600x actual speed).

## Supplementary Video 14: PenG rapidly stops constriction. mNeonGreen-PBP2B (ME7) cells imaged in a cellASIC device before and during treatment with excess PenG in fast growth conditions (rich media 37^o^C). Cells were imaged by ring-HiLO with 1 s exposure at 1 frame/min. Acquisitions displayed at 10 frames per second (600x actual speed).

**Supplementary Video 15: Effect of PC19 treatment on FtsZ-GFP cells in slow growth conditions.** FtsZ-GFP (SH130) cells imaged in a cellASIC device before and during treatment with excess PC19 in slow growth conditions (poor media 30^o^C). Cells were imaged by ring-HiLO with 1 s exposure at 1 frame/min. Acquisitions displayed at 10 frames per second (600x actual speed).

**Supplementary Video 16: Effect of PC58 treatment on FtsZ-GFP cells.** FtsZ-GFP (SH130) cells imaged in a cellASIC device before and after treatment with excess PC58 in fast growth conditions (rich media 37^o^C). Cells were imaged by ring-HiLO with 1 s exposure at 1 frame/min. Acquisitions displayed at 10 frames per second (600x actual speed).

**Supplementary Video 17: Effect of MciZ treatment on FtsZ-GFP cells.** FtsZ-GFP (SH130) cells imaged in a cellASIC device before and after treatment with 20 μM *B. subtilis* MciZ (Peptide Specialty Laboratories GmbH) in fast growth conditions (rich media 37^o^C). Cells were imaged by ring-HiLO with 1 s exposure at 1 frame/min. Acquisitions are displayed at 10 frames per second (600x actual speed).

**Supplementary Video 18: Effect of GTPase-deficient mutant FtsZ(D213A) expression on Z-ring condensation and constriction.** A strain expressing FtsZ-GFP from the native locus and the GTPase-deficient mutant FtsZ(D213A) from an inducible promoter (SH131) imaged on agarose pads containing 10 µM IPTG in slow growth conditions (poor media 30^o^C). Liquid cultures were induced with 10 µM IPTG for 1 hour prior to imaging. Cells were imaged by ring-HiLO with 1 s exposure at 1 frame/min. Acquisitions displayed at 10 frames per second (600x actual speed).

**Supplementary Video 19: PC19 treatment prevents mature rings with low PBP2B signal from constricting.** GFP-FtsZ HaloTag-PBP2B (SH212) cells labelled with JF549 HaloTag ligand imaged in a cellASIC device before and during treatment with excess PC19 in fast growth conditions (rich media 37^o^C). Representative mature ring with low PB2B signal (white circle) tracked manually using TrackMate. Cells were imaged by ring-HiLO with 1 s exposure at 1 frame/min. Acquisitions displayed at 10 frames per second (600x actual speed). GFP-FtsZ: green, JF549-HaloTag-PBP2B: magenta.

**Supplementary Video 20: Mature Z-rings with high PBP2B signal continue constricting post-PC19 treatment.** GFP-FtsZ HaloTag-PBP2B (SH212) cells labelled with JF549 HaloTag ligand imaged in a cellASIC device before and during treatment with excess PC19 in fast growth conditions (rich media 37^o^C). Representative mature ring with high PB2B signal (white circle) tracked manually using TrackMate. Cells were imaged by ring-HiLO with 1 s exposure at 1 frame/min. Acquisitions displayed at 10 frames per second (600x actual speed). GFP-FtsZ: green, JF549-HaloTag-PBP2B: magenta.

**Supplementary Video 21: Effect of PC19 treatment on mNeonGreen-PBP2B cells in fast growth conditions.** mNeonGreen-PBP2B (ME7) cells imaged in a cellASIC device before and during treatment with excess PC19 in fast growth conditions (rich media 37^o^C). Cells were imaged by ring-HiLO with 1 s exposure at 1 frame/min. Acquisitions displayed at 10 frames per second (600x actual speed).

**Supplementary Video 22: Effect of PC19 treatment on mNeonGreen-PBP2B cells in slow growth conditions.** mNeonGreen-PBP2B (ME7) cells imaged in a cellASIC device before and during treatment with excess PC19 in slow growth conditions (poor media 30^o^C). Cells were imaged by ring-HiLO with 1 s exposure at 1 frame/min. Acquisitions displayed at 10 frames per second (600x actual speed).
