# Supplementary figures and images for: FtsZ treadmilling is essential for Z-ring condensation and septal constriction initiation in Bacillus subtilis cell division
Source: Nat Commun. 2021 Apr 27;12:2448. doi: 10.1038/s41467-021-22526-0 (PMC8079713; doi:10.1038/s41467-021-22526-0)

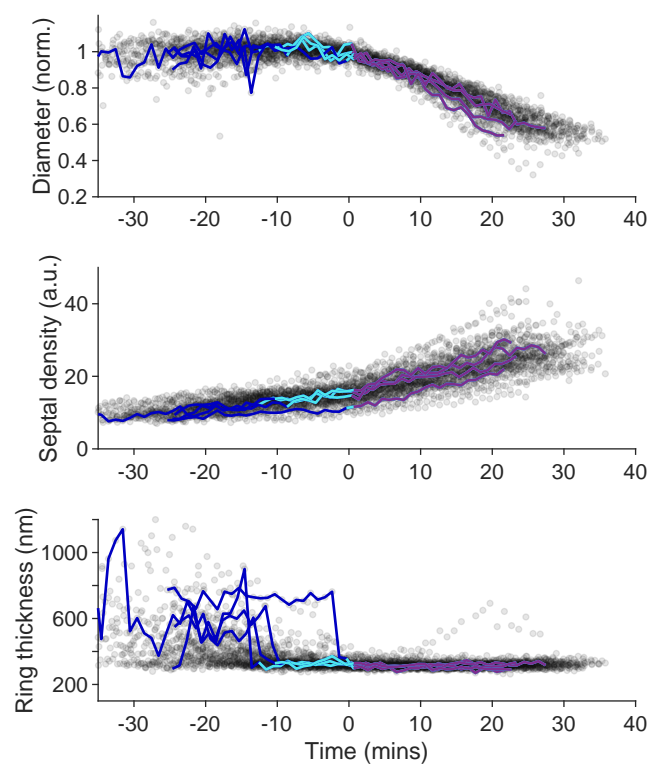

Supplement: Supplementary file 27 — Source Data [file 41467_2021_22526_MOESM27_ESM.zip › Whitley2020_source_data/Fig1/ringDynamics-lineStateColor.pdf]

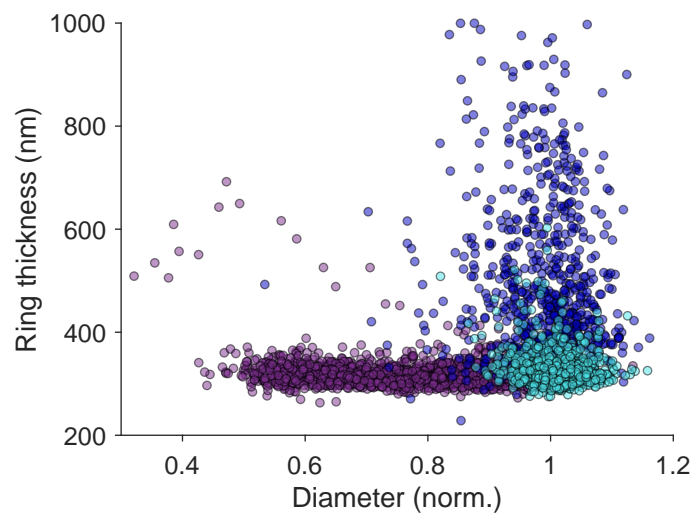

Supplement: Supplementary file 27 — Source Data [file 41467_2021_22526_MOESM27_ESM.zip › Whitley2020_source_data/Fig1/Ring_thickness-diameterNorm.pdf]

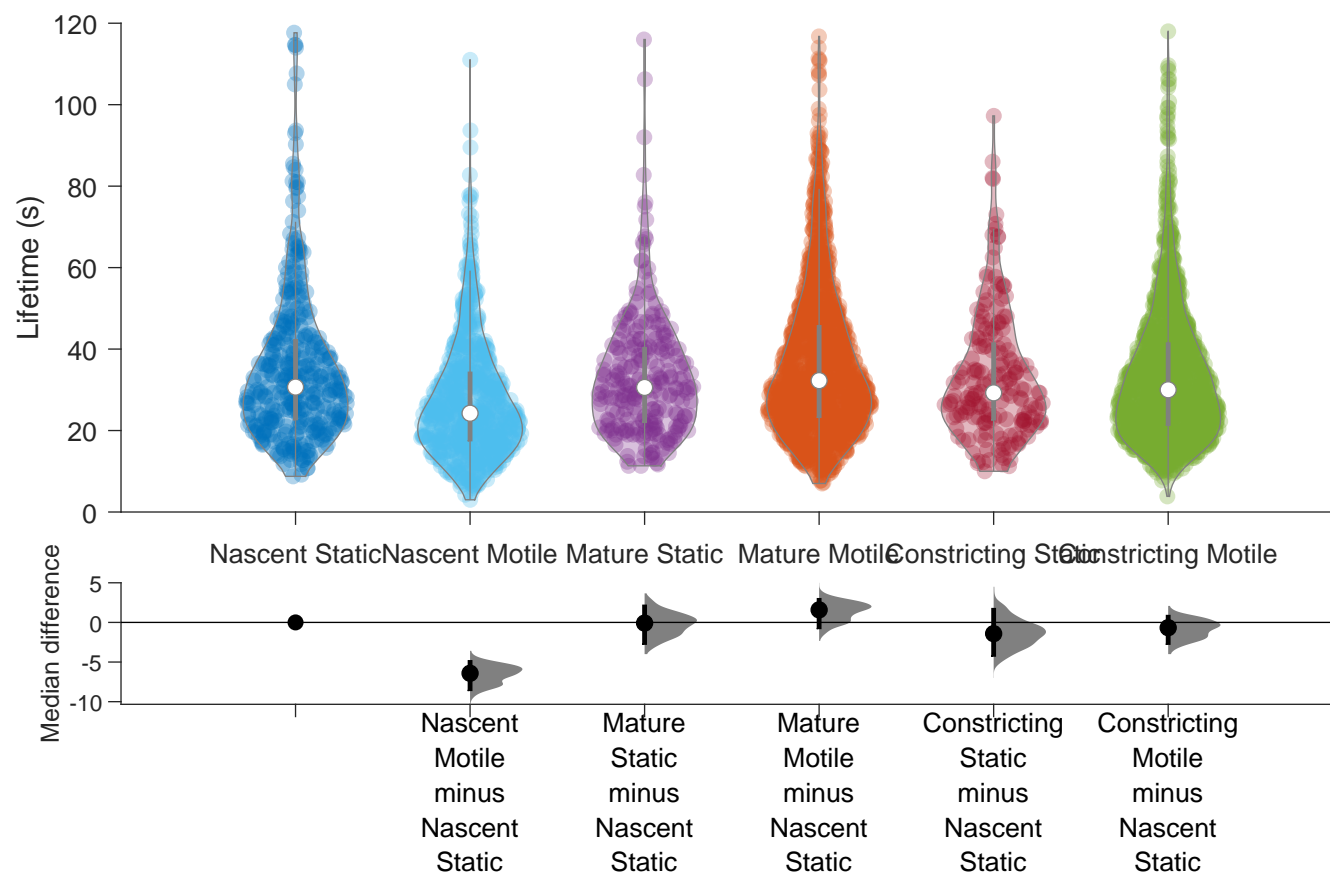

Supplement: Supplementary file 27 — Source Data [file 41467_2021_22526_MOESM27_ESM.zip › Whitley2020_source_data/SuppFig11/z-lifetime-static-motile-dabest.pdf]

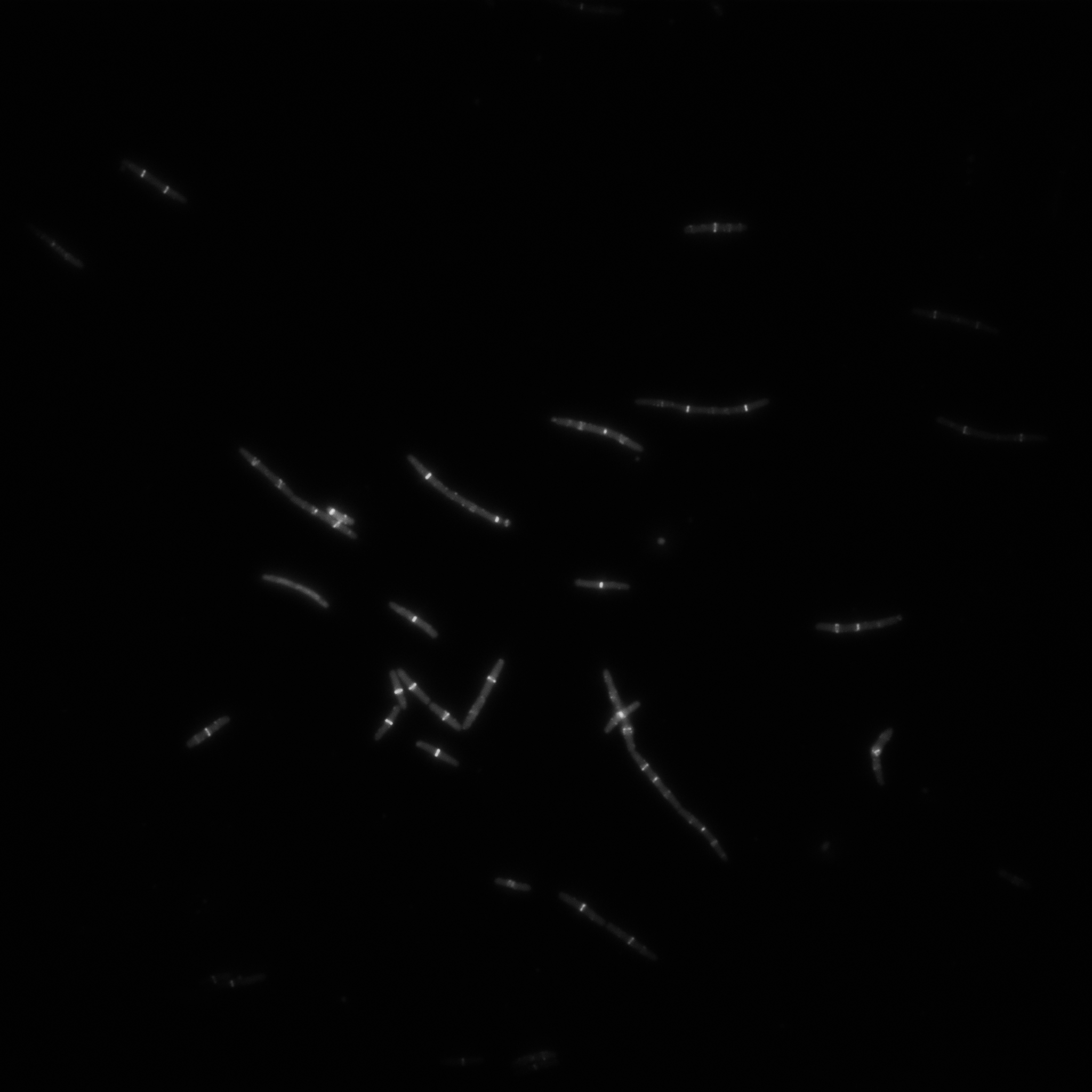

Supplement: Supplementary file 27 — Source Data [file 41467_2021_22526_MOESM27_ESM.zip › Whitley2020_source_data/SuppFig18/SIfig18e/SH130/slide2_pos15_488_MMStack_Default.ome.tif]

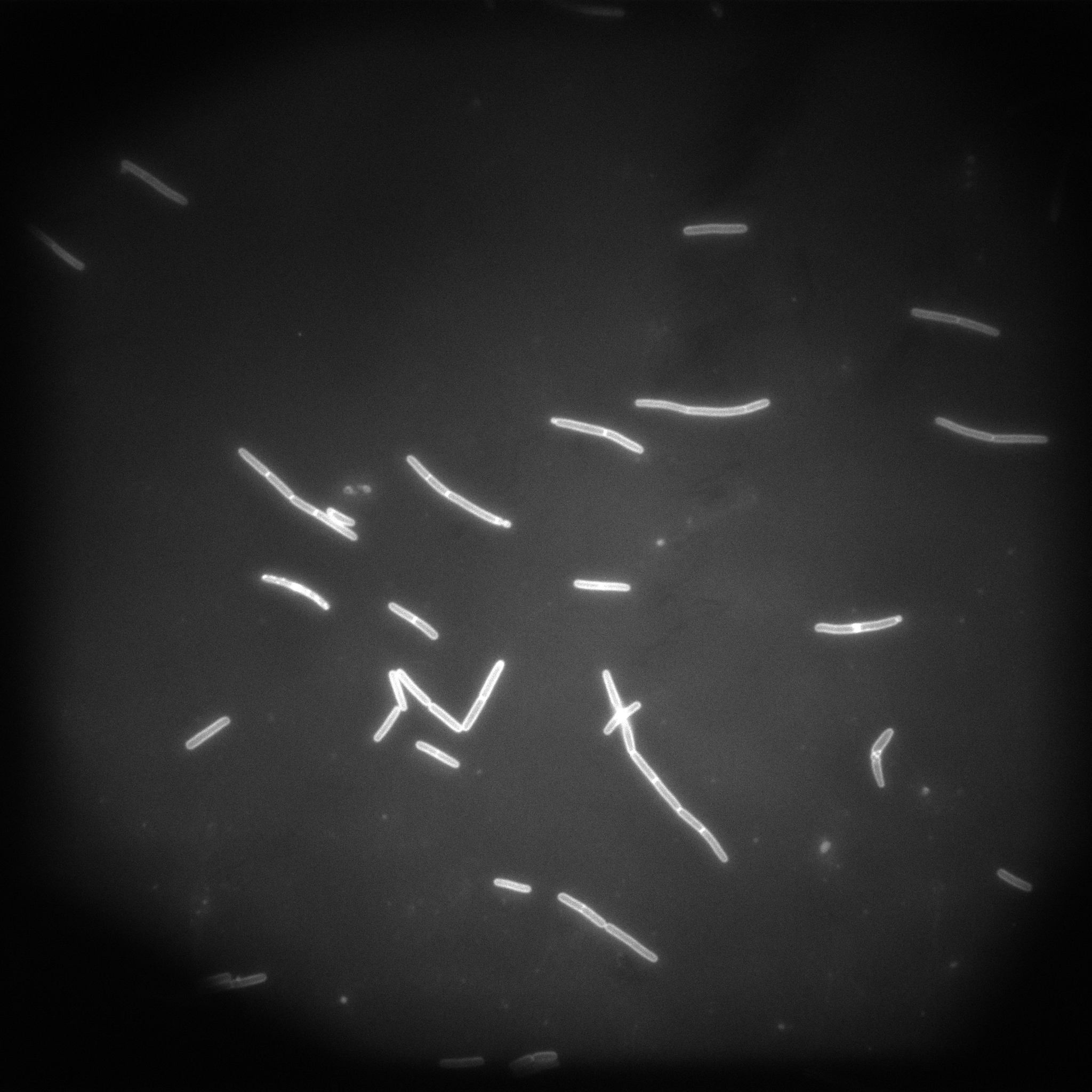

Supplement: Supplementary file 27 — Source Data [file 41467_2021_22526_MOESM27_ESM.zip › Whitley2020_source_data/SuppFig18/SIfig18e/SH130/slide2_pos15_561_MMStack_Default.ome.tif]

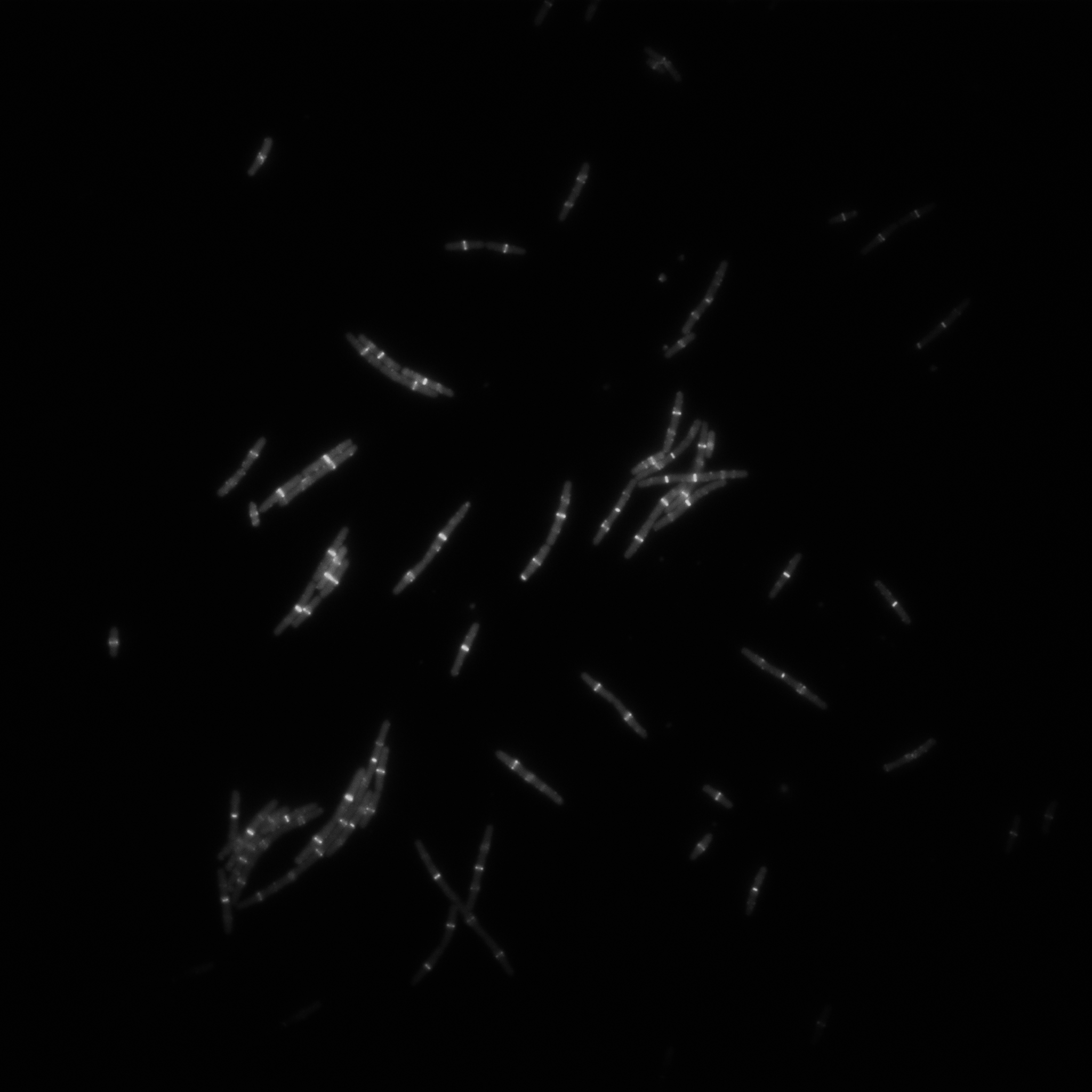

Supplement: Supplementary file 27 — Source Data [file 41467_2021_22526_MOESM27_ESM.zip › Whitley2020_source_data/SuppFig18/SIfig18e/SH130/slide2_pos19_488_MMStack_Default.ome.tif]

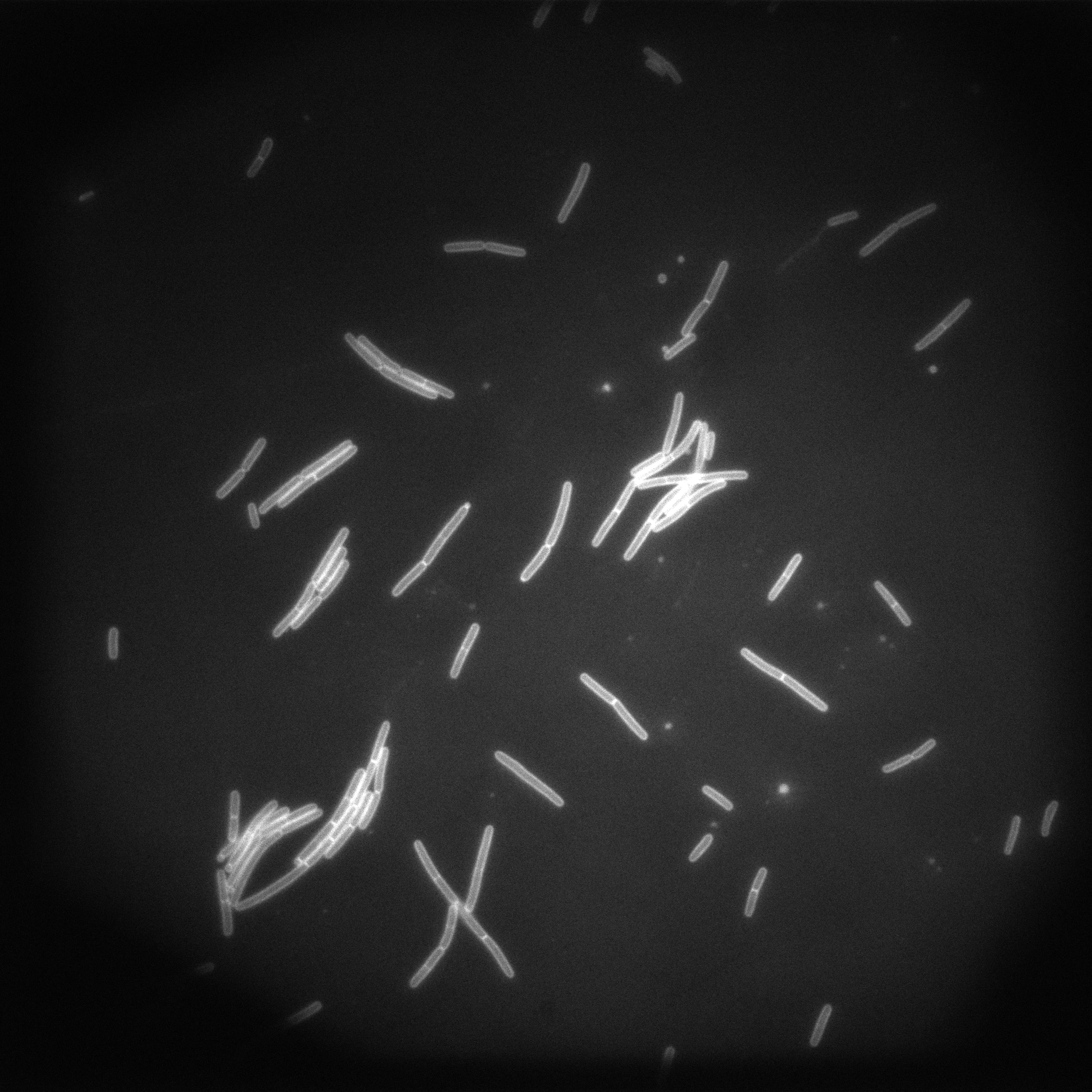

Supplement: Supplementary file 27 — Source Data [file 41467_2021_22526_MOESM27_ESM.zip › Whitley2020_source_data/SuppFig18/SIfig18e/SH130/slide2_pos19_561_MMStack_Default.ome.tif]

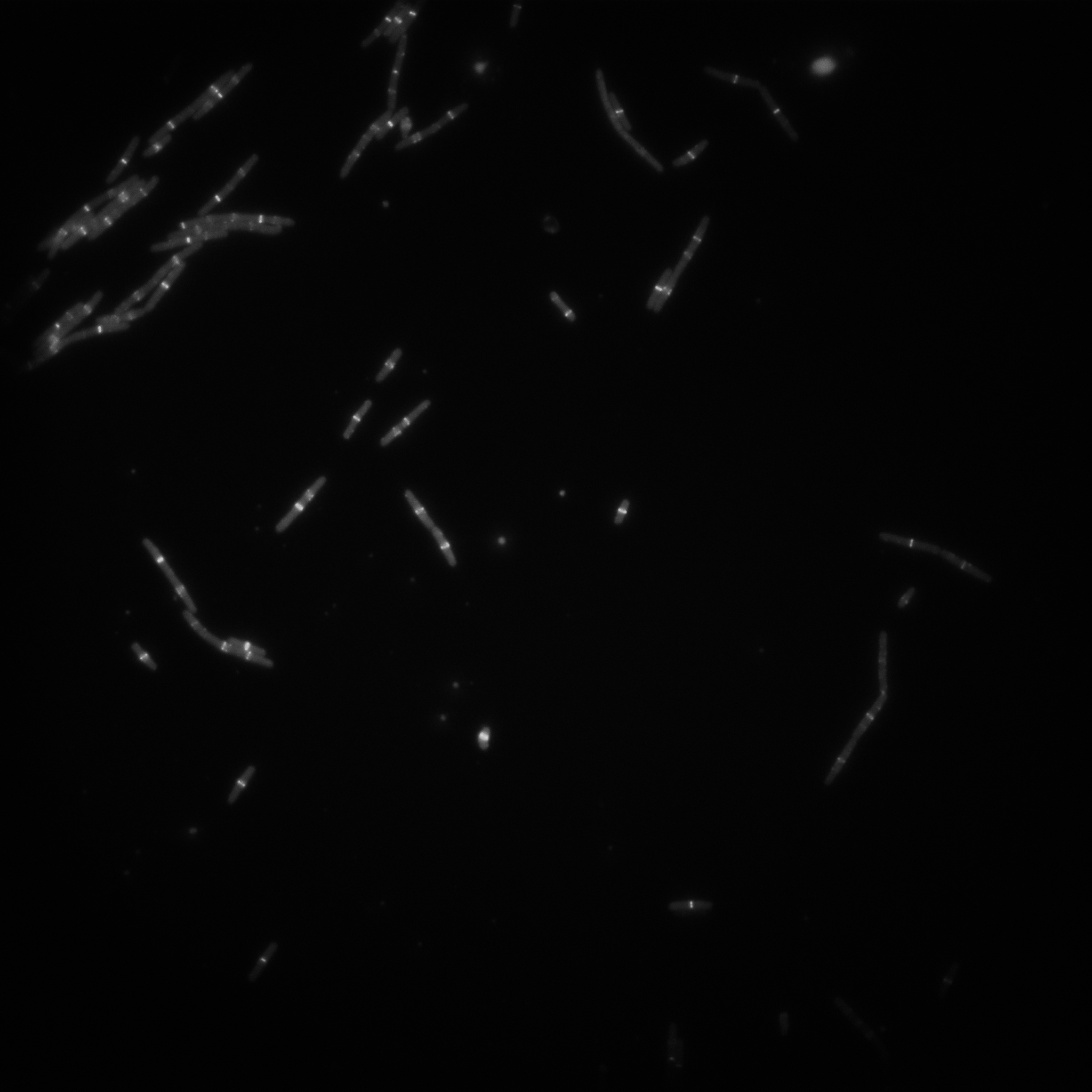

Supplement: Supplementary file 27 — Source Data [file 41467_2021_22526_MOESM27_ESM.zip › Whitley2020_source_data/SuppFig18/SIfig18e/SH130/slide2_pos8_488_MMStack_Default.ome.tif]

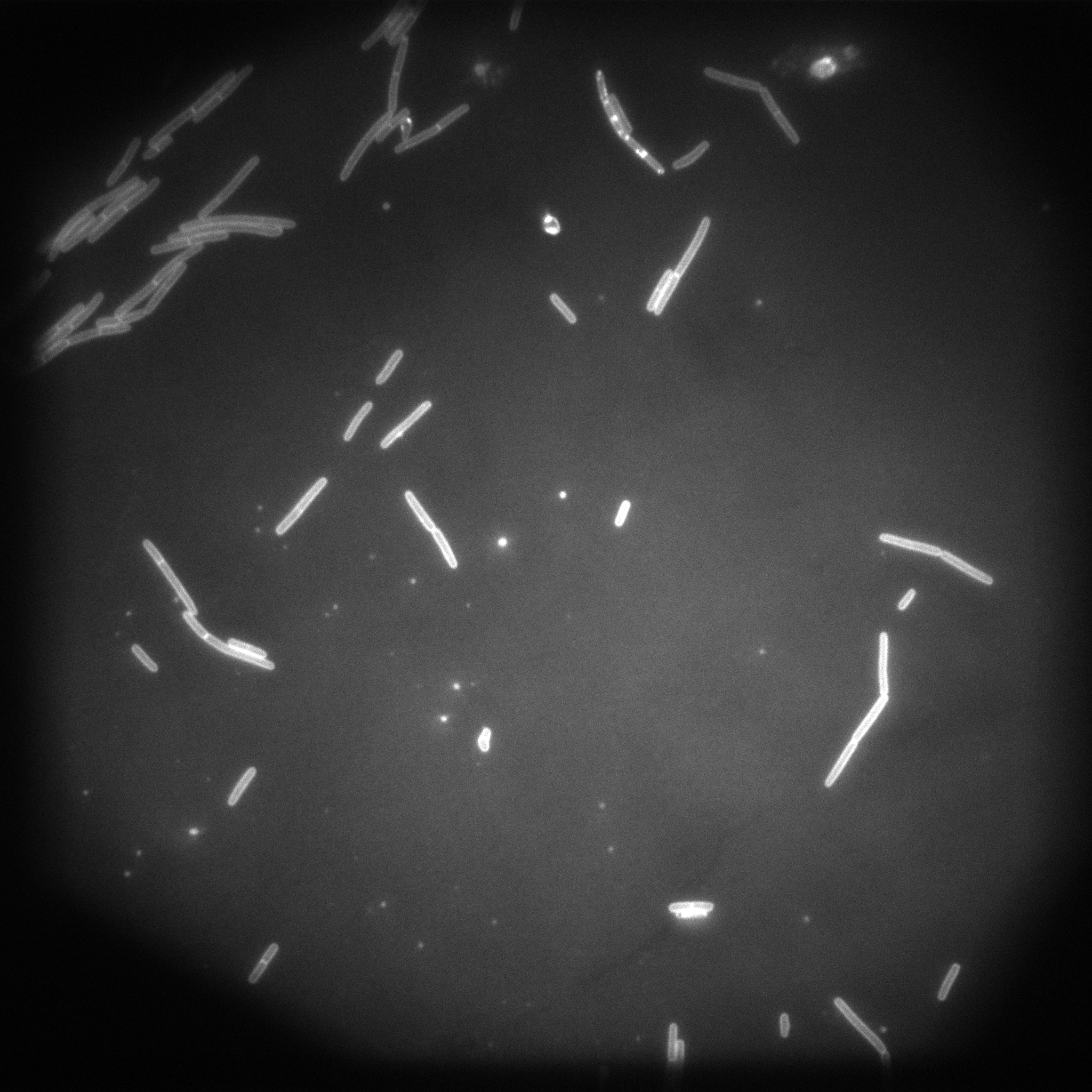

Supplement: Supplementary file 27 — Source Data [file 41467_2021_22526_MOESM27_ESM.zip › Whitley2020_source_data/SuppFig18/SIfig18e/SH130/slide2_pos8_561_MMStack_Default.ome.tif]

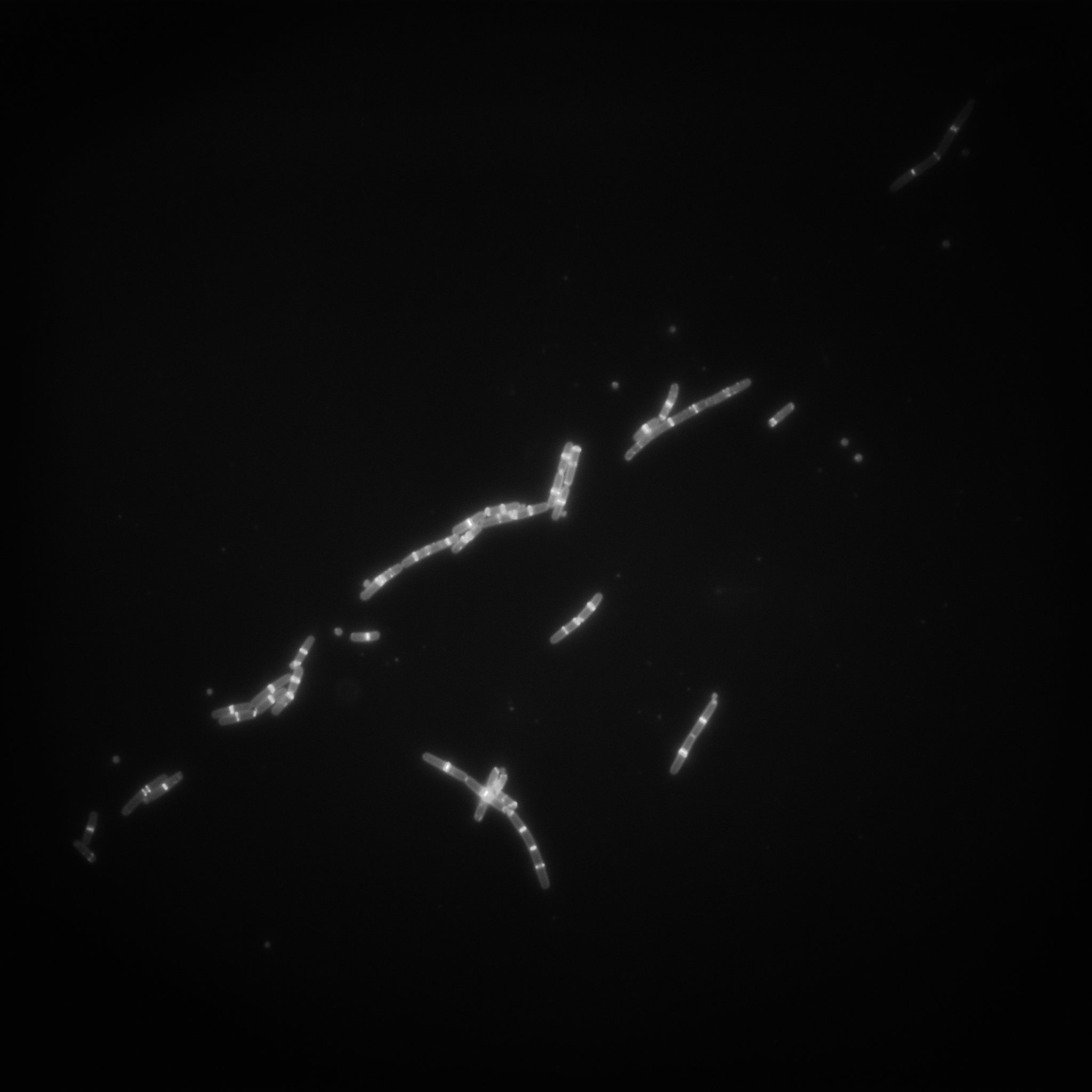

Supplement: Supplementary file 27 — Source Data [file 41467_2021_22526_MOESM27_ESM.zip › Whitley2020_source_data/SuppFig18/SIfig18e/SH131/pos2_488_MMStack_Default.ome.tif]

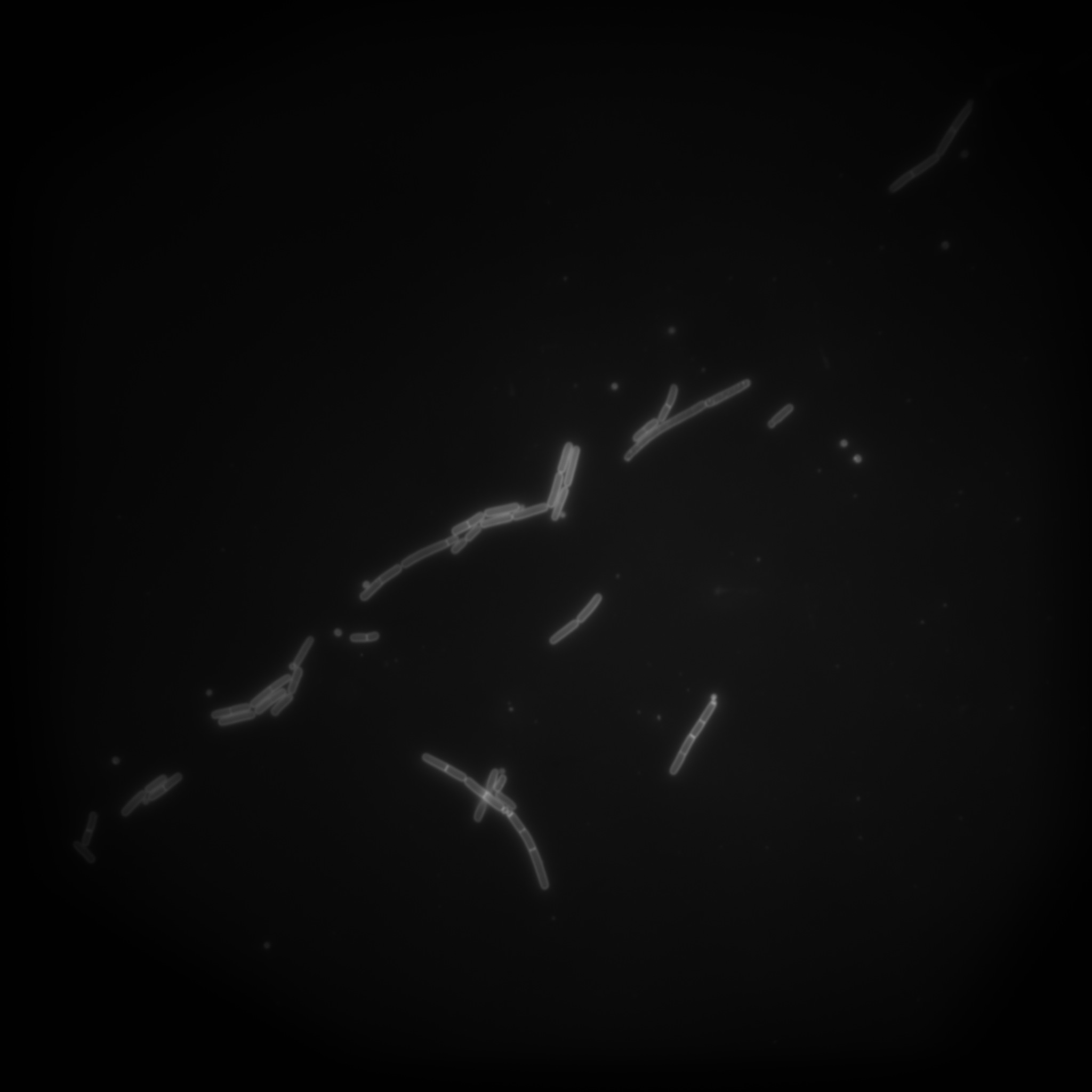

Supplement: Supplementary file 27 — Source Data [file 41467_2021_22526_MOESM27_ESM.zip › Whitley2020_source_data/SuppFig18/SIfig18e/SH131/pos2_561_MMStack_Default.ome.tif]

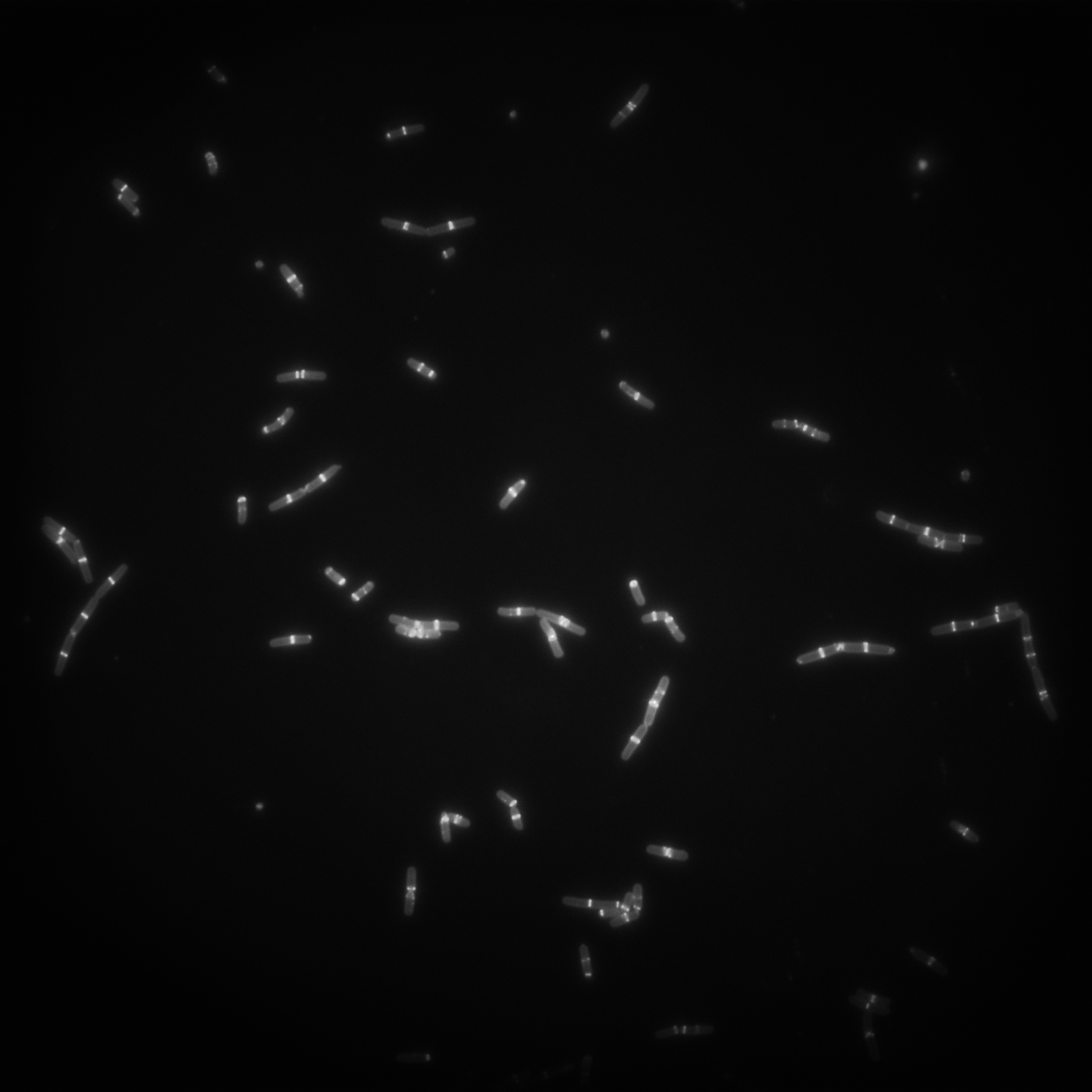

Supplement: Supplementary file 27 — Source Data [file 41467_2021_22526_MOESM27_ESM.zip › Whitley2020_source_data/SuppFig18/SIfig18e/SH131/pos3_488_MMStack_Default.ome.tif]

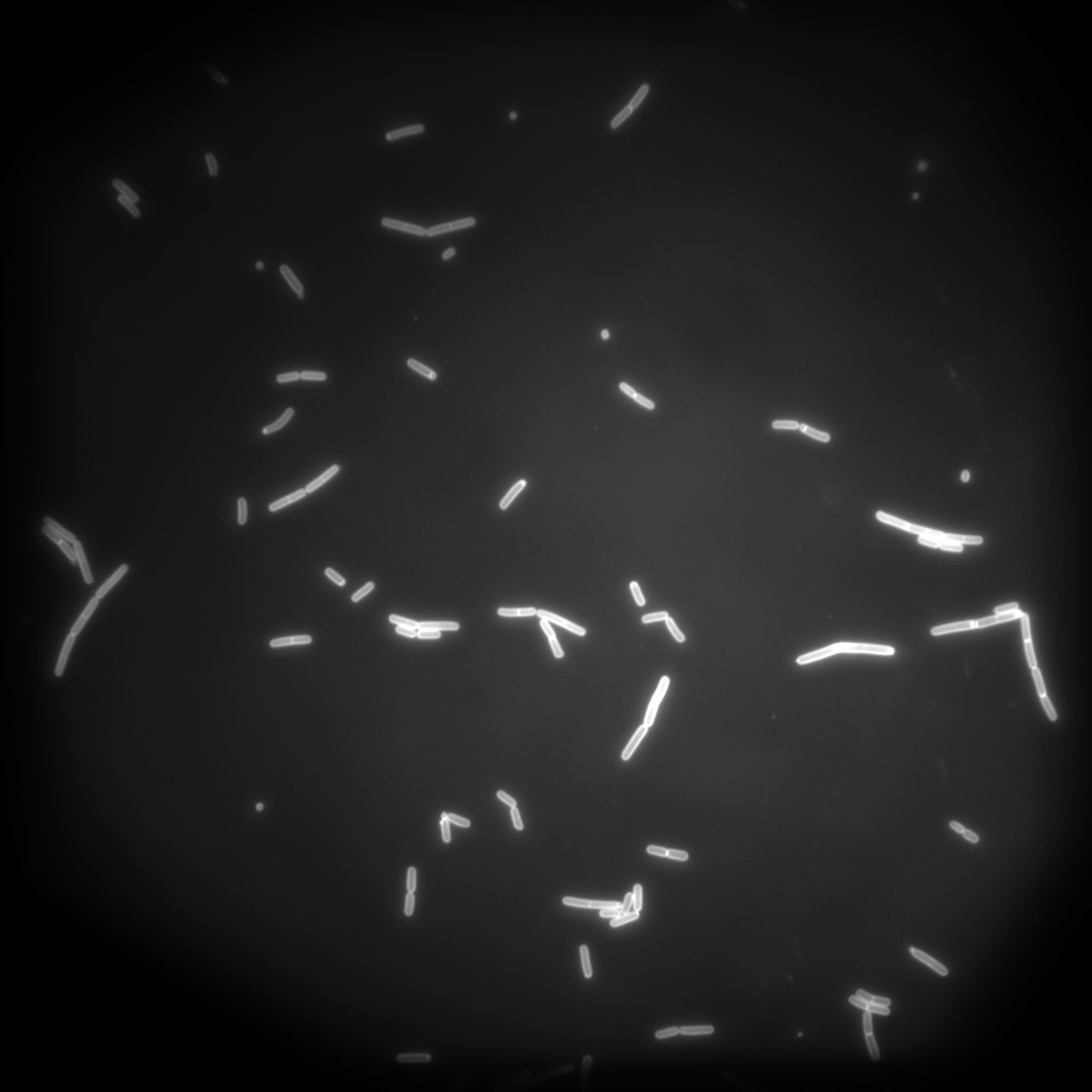

Supplement: Supplementary file 27 — Source Data [file 41467_2021_22526_MOESM27_ESM.zip › Whitley2020_source_data/SuppFig18/SIfig18e/SH131/pos3_561_MMStack_Default.ome.tif]

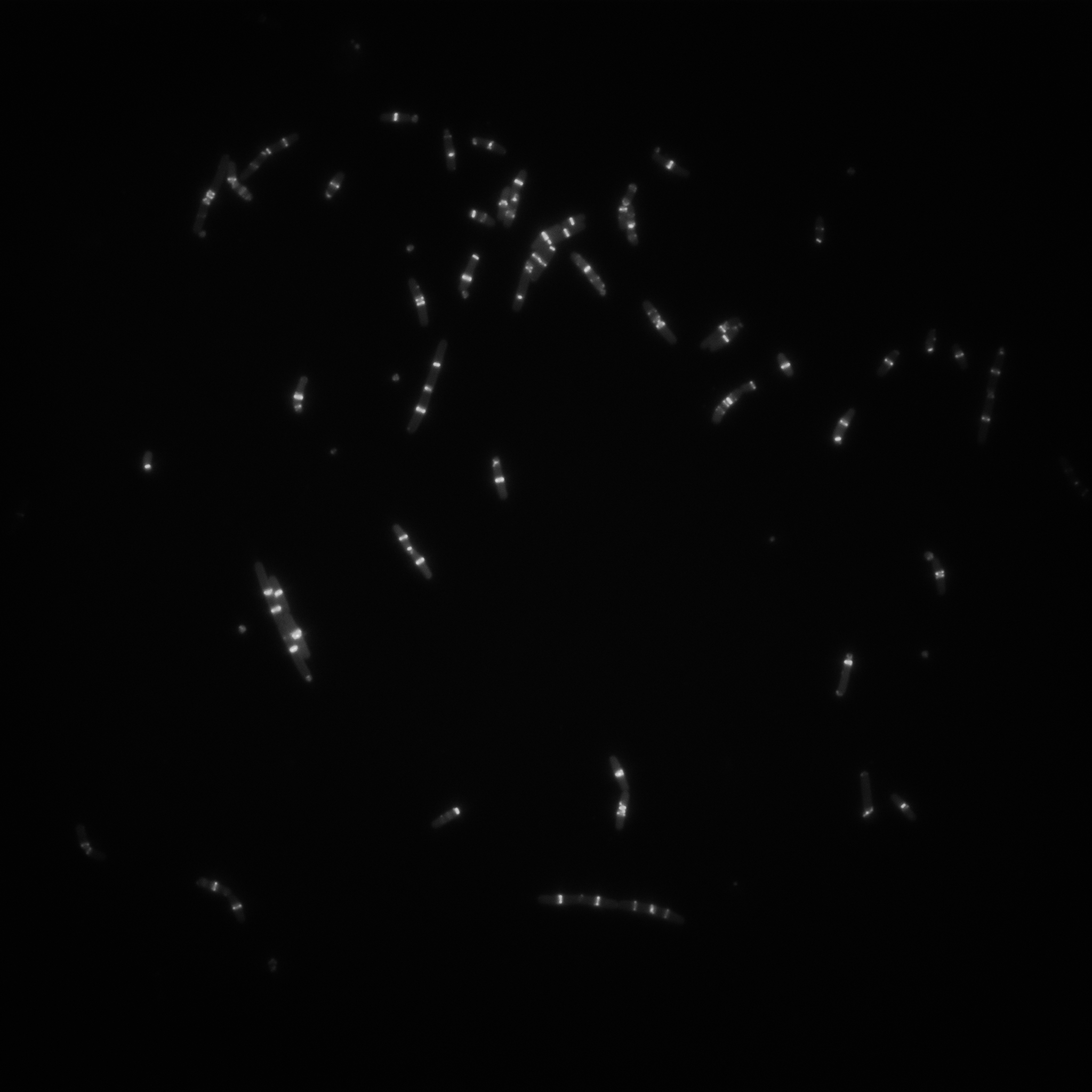

Supplement: Supplementary file 27 — Source Data [file 41467_2021_22526_MOESM27_ESM.zip › Whitley2020_source_data/SuppFig18/SIfig18e/SH131/pos9_488_MMStack_Default.ome.tif]

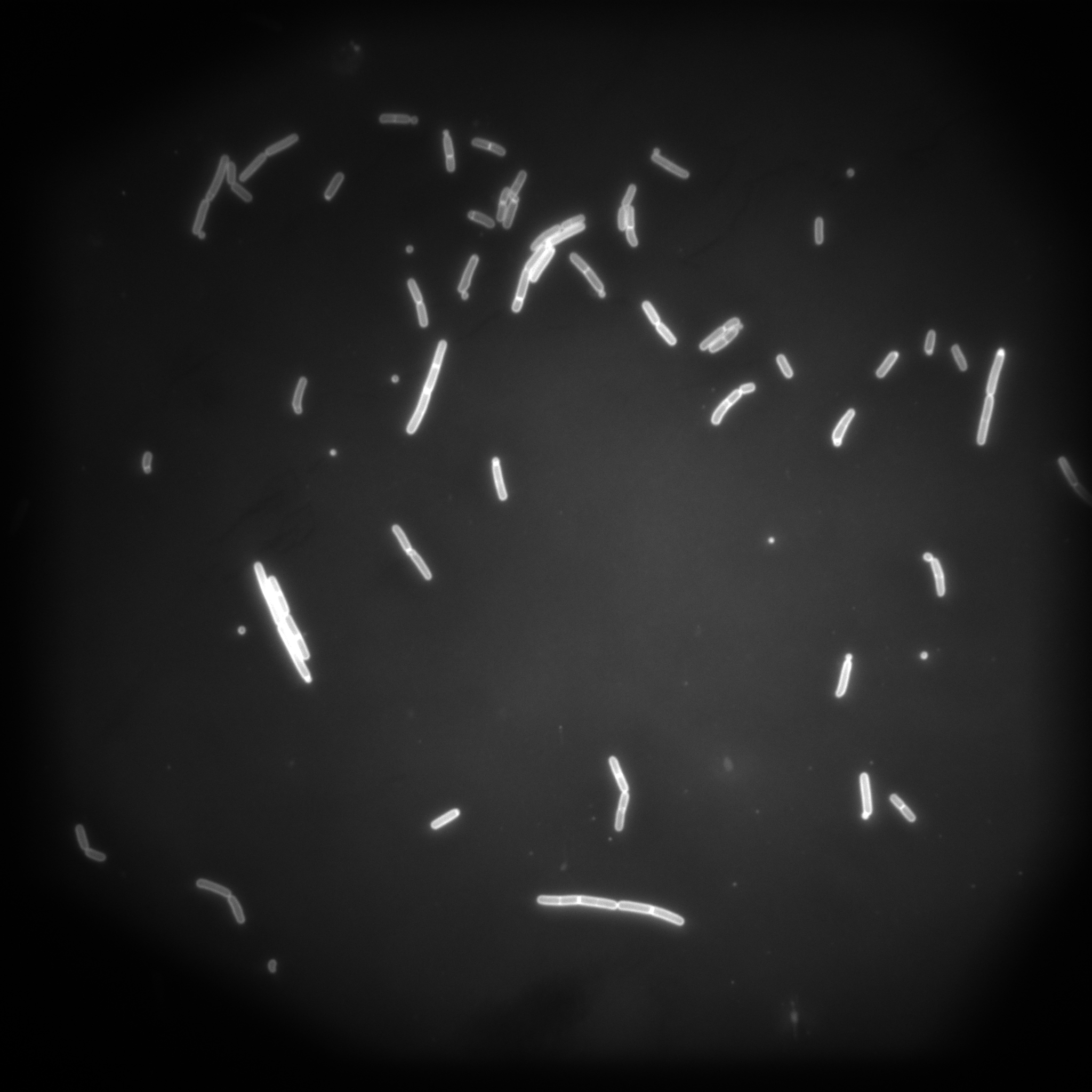

Supplement: Supplementary file 27 — Source Data [file 41467_2021_22526_MOESM27_ESM.zip › Whitley2020_source_data/SuppFig18/SIfig18e/SH131/pos9_561_MMStack_Default.ome.tif]

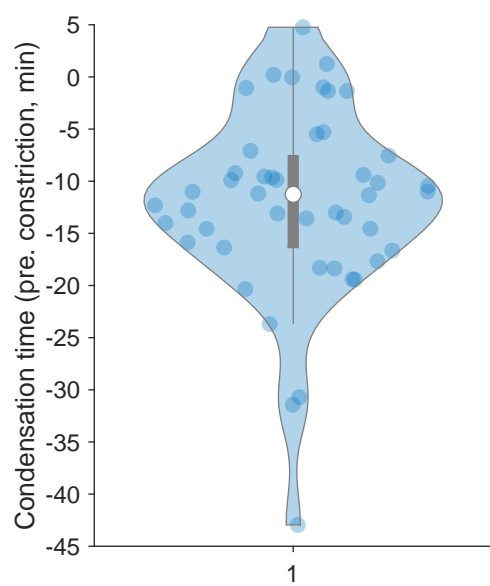

Supplement: Supplementary file 27 — Source Data [file 41467_2021_22526_MOESM27_ESM.zip › Whitley2020_source_data/SuppFig4/Z-condensation-time.pdf]

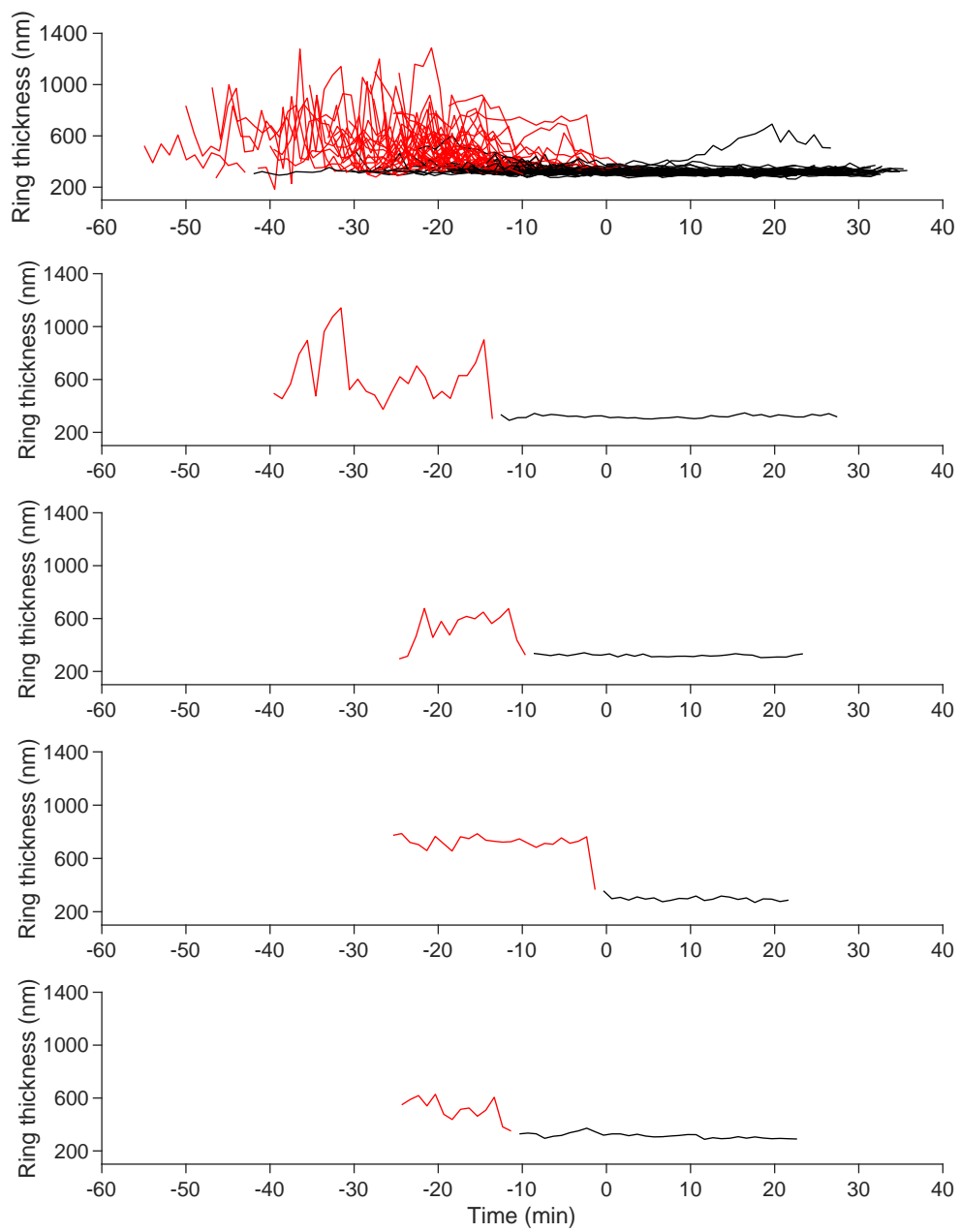

Supplement: Supplementary file 27 — Source Data [file 41467_2021_22526_MOESM27_ESM.zip › Whitley2020_source_data/SuppFig4/Z-condensation-tracks.pdf]

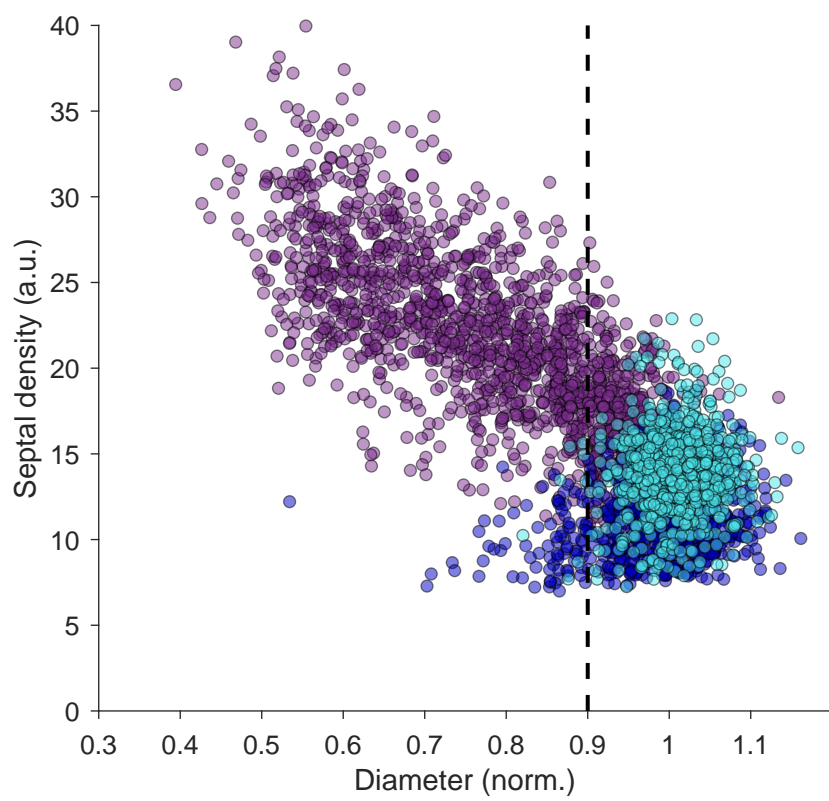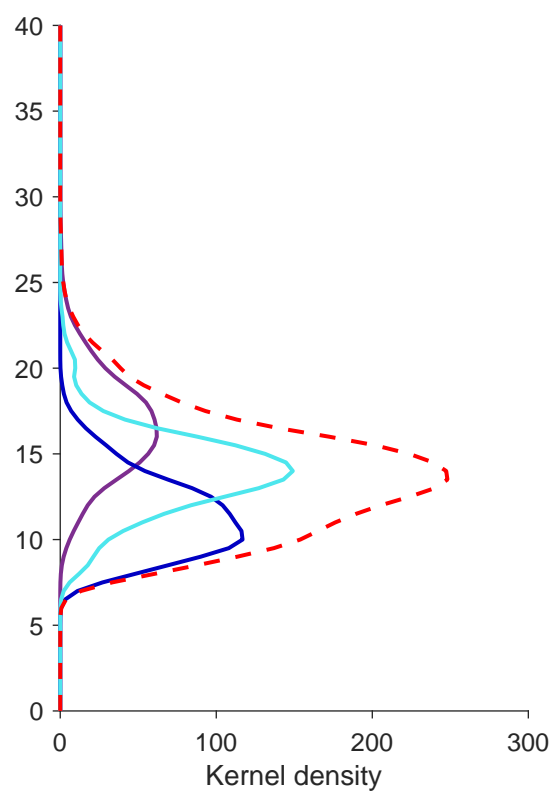

Supplement: Supplementary file 27 — Source Data [file 41467_2021_22526_MOESM27_ESM.zip › Whitley2020_source_data/SuppFig8/Ring_density-diameterNorm-kdeSide.pdf]
